# Toxic Effects of Trichloroethylene on Rat Neuroprogenitor Cells

**DOI:** 10.3389/fphar.2018.00741

**Published:** 2018-07-10

**Authors:** Mohamed M. Salama, Doaa A. El-Naggar, Rania H. Abdel-Rahman, Seham A. G. Elhak

**Affiliations:** ^1^Forensic Medicine and Clinical Toxicology Department, Faculty of Medicine, Mansoura University, Mansoura, Egypt; ^2^Medical Experimental Research Centre, Faculty of Medicine, Mansoura University, Mansoura, Egypt

**Keywords:** trichloroethylene, neurotoxicity, neurospheres, *in vitro*, developmental

## Abstract

Trichloroethylene (TCE) is a common volatile organic solvent which is considered as an ubiquitous environmental pollutant. It is claimed to be a developmental neurotoxicant. Our group evaluated previously its impact on three-dimensional neurospheres *in vitro*. The current work aims to investigate the neurotoxic effects of a lower concentration of TCE on the same system. To perform the experiment, neural progenitor cells were obtained from the brains of nine newborn rats. Afterward, these cells were cultured in both growth and differentiation media to get the neurospheres. Cell cultures were divided into two groups: group 1 (control), group 2 (exposed to 0.25 μM TCE). Neurospheres were photographed at different durations and assessment of the morphological changes such as proliferation and differentiation of neurospheres was done. In addition, cell viability, apoptosis, and necrosis were analyzed using flow cytometry to clarify the mechanism of involved cytotoxicity. The results revealed that TCE-treated neurospheres showed significantly decreased proliferation on days 7 and 14. These cells failed to show the neurogenic differentiation seen in the neurospheres of the control group. Furthermore, a highly significant decrease in viability and a significant increase in the number of apoptotic cells were observed in the treated cells in comparison to the control group. The present work confirmed that TCE, at very low doses relevant to daily life exposure in humans, caused neurotoxic effects in 3D neurosphere model through the affection of neural proliferation and differentiation as well as disturbance of cell viability and apoptosis.

## Introduction

There is a strong evidence indicating that environmental exposure to various chemicals at critical developmental stages affects the behavioral and neurological development in children (De Felice et al., 2015). It is hypothesized that developmental neurotoxicity (DNT) results from disturbance of some biological processes, such as differentiation, proliferation, apoptosis, and neurite growth (Bal-Price et al., 2012; Kadereit et al., 2012).

Trichloroethylene (TCE) is a common volatile organic solvent that has been widely used in industrial applications and consumer or commercial products such as a cleaning and degreasing agent in ink and varnishes. It is also a common environmental pollutant usually found in air, soil, and water. Many studies have shown that TCE is associated with serious health hazards as well as neurodevelopmental abnormalities due to gestational exposure to this chemical (Environmental Protection Agency [EPA], 2014).

Accordingly, TCE is highly suggested to be classified as a developmental neurotoxicant. However, this claim needs to be validated by more detailed DNT testing such as the three-dimensional (3D) neurosphere system. This model represents an *in vivo*-like microenvironment which could reflect the basic developmental processes of the growing brain and improved the ability to verify the neurotoxic effects of chemicals during early life exposure (Choi et al., 2013; Dingle et al., 2015).

The mechanism by which developmental exposure to TCE induces neurotoxicity is still unclear (Blossom et al., 2017). In a previous work, our group tested the neurotoxicity of a higher dose of TCE (1 μM) which is claimed to be safe in human (Abdraboh et al., 2017). In the present work, we investigate the neurotoxic effects of a much lower dose of TCE (0.25 μM) on the same system of 3D neurospheres.

## Materials and Methods

The present work was approved by the Institutional Review Board, Faculty of Medicine, Mansoura University (code MS/896). Nine newborn rats (1 day after birth), Sprague Dawley strain, regardless of sex, were obtained from the animal house of Medical Experimental Research Center (MERC). All chemicals and reagents were purchased from Sigma-Aldrich Company, St. Louis, MO, United States unless declared otherwise.

Cell culture media included the following: (a) Growth medium: Dulbecco’s modified Eagle medium and Ham’s F-12 (1:1 MIX) (Lonza, cat. no. BE12-719F, Basel, Switzerland) supplemented with 10% Fetal bovine serum “FBS” (Hyclone, San Angelo, TX, United States), 1% L-glutamine (Gibco, Carlsbad, CA, United States) and 1% Penicillin–streptomycin–Amphotericin B Mixture (Lonza, cat. no. 17-745E, Basel, Switzerland). (b) Differentiation medium: [Dulbecco’s modified Eagle medium and Ham’s F12 (3:1) enriched with neural growth factors 1% B27, 1% N2 supplement, 20 ng/mL Recombinant human Fibroblast growth factor basic (Invitrogen/Gibco, Carlsbad, CA, United States), 2–5% FBS and 1% Penicillin–streptomycin–Amphotericin B Mixture].

### Preparation of Neurospheres

Neurospheres were obtained as described previously (Abdraboh et al., 2017) by aseptically dissecting out the cortices from the brains of nine newborn rats and isolating rat neural progenitor cells. In brief, the cortices were chopped into very tiny pieces on separate sterilized Petri dishes. Then, 10 ml trypsin EDTA solution were added to each tissue and incubated for 45 min at 37°C with constant shaking. To inactivate trypsin, 10 ml of growth media were added to each sample, and pipetted up and down 10 more times. Then, the brain tissues were filtered using sterilized mesh filter. Cell suspensions (1 × 10^6^ cells) were transferred to disposable conical tubes and centrifuged at 2000 RPM for 10 min to precipitate the pellets. Each pellet was resuspended in 15 ml growth medium and transferred into a tissue culture flask in humidified 5% CO_2_ incubator at 37°C for 24–48 h (Iwamaru et al., 2013; Louis et al., 2013).

### Trichloroethylene Exposure

Trichloroethylene (cat. no. 79-01-6): (density: 1.463 g/mL, purity: ≥99%, Technical grade) was obtained. Thereafter, the nine tissue culture flasks containing the neurospheres were randomly divided into two groups; Group 1: neurospheres served as a control group and received no treatment. Group 2: neurospheres were treated with 0.25 μM trichloroethylene (Environmental Protection Agency [EPA], 2014).

### Assessment of the Effects of TCE on Neurospheres

It was performed by 40× objective through evaluation of three neurospheres per field in eight randomly distributed visual fields per culture well in at least three biological replicates per concentration in a blind manner.

#### Cell Proliferation

The neurospheres were photographed at different durations (0, 3, 7, and 14 days). Then, each sphere size was determined by software analyses (Cell Profiler, version 2.1; Broad Institute, freely downloaded from http://www.cellprofiler.org). The diameter of each neurosphere was measured in μm and exported to excel file further to statistical analysis.

#### Cell Differentiation

Images of the plated neurospheres were evaluated regarding distinct neuronal morphology with fasciculation of neurites that radiate from the central aggregation of neuronal perikarya. In addition, the cell capacity to differentiate into dopaminergic neurons was challenged. It was then assessed through immunostaining against anti-tyrosine hydroxylase (TH) antibody (Novus Biologicals, United States, 1:200 dilution: Cat.No. #NB300–109).

#### Detection of Cell Viability, Necrosis, and Apoptosis by Flow Cytometry (Wlodkowic et al., 2010)

• Initially, cell viability was estimated using Trypan Blue Exclusion test. Thereafter, to evaluate the aforementioned parameters more precisely, Annexin V kit, Propidium Iodide and 1X Binding Buffer (cat. No. 556547 BD Pharmingen FITC “fluorescein isothiocyanate” apoptosis Kit, Princeton, NJ, United States) were used.
• The cells were washed twice with cold PBS and then resuspended in 1X Binding Buffer. Five μl Annexin V (FITC label) and 5 μl Propidium Iodide (PI) were added. Gently, the cells were mixed using vortex and incubated for 15 min at room temperature (25°C) in the dark. 200 μl of 1X Binding Buffer was added. The cells were evaluated for the cell cycle by Flow Cytometer (BD Accuri^TM^ C6, Piscataway, NJ, United States) within 1 h. The intact membrane of living cells excludes cationic dyes, such as PI which can stain the nucleus in case of lost membrane integrity while Annexin V (apoptotic marker) can bind to the phosphatidylserine present on the surface of apoptotic cells. In early apoptosis, phosphatidyl serine is exposed on the cell surface which is detected by Annexin V. Due to their extensive membrane damage; necrotic cells are quickly and brightly stained with PI and will appear as a peak at very high fluorescence values. Apoptotic cells will be dimly stained and show a much lower uptake of PI than that seen with necrotic cells.
• Cell death was evaluated as follows: dot plots were generated and divided into four quadrants (UR, upper right; UL, upper left; LR, lower right; LL, lower left). The LL quadrant represented the living non-apoptotic cells (negative for both annexin V and PI). The living early apoptotic cells were shown in the LR quadrant (annexin V positive cells but negative for PI). The UL quadrant demonstrated the necrotic cells (negative to annexin V and positive for PI). Whereas, the late apoptotic cells were shown in the UR quadrant (both annexin V and PI positive cells).

### Statistical Analysis

Data were assessed for normality using Shapiro–Wilk test, then statistical analyses were performed. Differences between mean values were assessed for statistical significance using a two-tailed Student’s *t*-test (GraphPad Prism 5.0 software, La Jolla, CA, United States). For all tests, *P*-value 0.05 was deemed significant.

## Results and Discussion

In the present work, we assessed how the rat neural progenitor cells were affected by TCE in a minimal dose (0.25 μM) at different timelines. The morphological changes such as proliferation and differentiation of neurospheres were evaluated to validate the neurotoxic effects of TCE. In addition, to clarify the mechanism of involved cytotoxicity, the cell viability, apoptosis, and necrosis were analyzed using flow cytometry.

Noteworthy, we utilized a higher concentration of TCE (1 μM) in a previous report (Abdraboh et al., 2017). This dose was proved to cause a significant time-dependent reduction in the proliferative capacity of neurospheres, failure of the cells to differentiate into astrocytes as well as a significant decrease of cell viability at 1 and 2 weeks duration. These findings could be extrapolated to the human population as the minimal occupational exposure among degreasing workers was found to be about (131 mg/m^3^) which is equivalent to 1 μM TCE (Environmental Protection Agency [EPA], 2014). Also, this concentration is the representative dose of the environmental concentration of TCE on surface water (1 μg/L) according to United States Environmental Protection Agency report (Agency for Toxic Substances and Disease Registry [ATSDR], 2016).

In the present study, we tried to assess the neurotoxic effect of a much lower dose of TCE than the previously investigated (Abdraboh et al., 2017). Based on a preliminary pilot study, we chose a TCE concentration of 0.25 μM that is equivalent to the average daily life human exposure occurring through inhalation and ingestion which is suggested to be 33 μg per day (Environmental Protection Agency [EPA], 2014).

**FIGURE 1:**
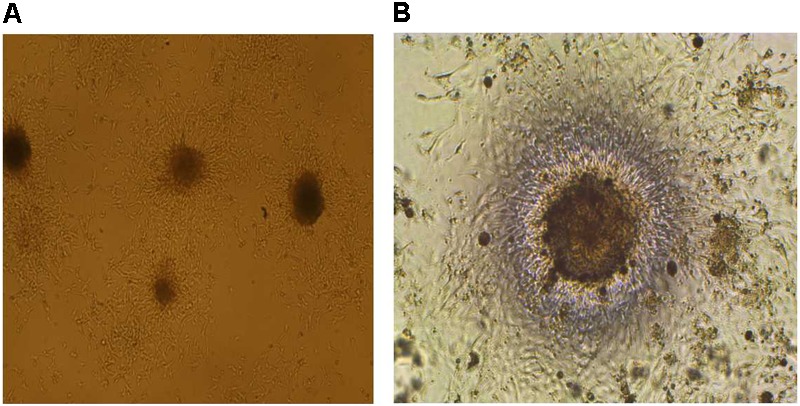
Image illustrating the growing neurospheres after 3 days of the study using low **(A)** and high **(B)** magnification.

Neurospheres were obtained by the same protocol described in our previously published work (Abdraboh et al., 2017). **Figure 1** shows the growing 3D neurospheres after 3 days. Then, the effects of TCE on the proliferation of neurospheres were evaluated through measurement of the cell diameter and assessment of the sequence of its increase as well as their size variation with the progress of time in the culture medium as illustrated in **Table 1**. The present findings reveal a normal pattern of proliferation and progressive increase in the diameter of the cells in the control group throughout the study period (from day 0 till day 14). Whereas, TCE-exposed neurospheres show reduced proliferation as evidenced by the decreased cell diameter.

**Table 1:** Neurospheres diameter (proliferation) in the studied groups.

Groups/Day	Group 1 (control)	Group 2 (TCE-treated group) (TCE: 0.25 μM)
0	161 ± 22.3	165 ± 30.1
3	299 ± 26.5	281 ± 39.7
7	642 ± 18.9	418 ± 27.7^∗^
14	812 ± 27.8	402 ± 23.5^∗^

*N.B. TCE, trichloroethylene. All values were expressed as Mean ± SD. ^∗^Statistically significant at *P* < 0.05 (between control and test groups).*

More or less similar, those results reported by Wang et al. (2001) who tested the vaporous toxicity of TCE (20–80 μl) on Chinese hamster ovarian (CHO-K1) cells (Wang et al., 2001). They found that there was a dose-dependent decrease in cellular proliferation. They suggested that proliferation arrest was dependent on GSH metabolism.

Interestingly, neurite outgrowth is relevant to study the DNT of chemicals as it is a critical process occurring during the development of the nervous system, which when disrupted, this may lead to serious adverse neurodevelopmental disorders (Harrill et al., 2013; Krug et al., 2013). Hence, neurite outgrowth could serve as a preliminary assessment tool for neuronal differentiation (Ryan et al., 2016). In addition, for more distinctive assessment of neurosphere differentiation, the capacity of the cells to differentiate into dopaminergic neurons was investigated.

**FIGURE 2:**
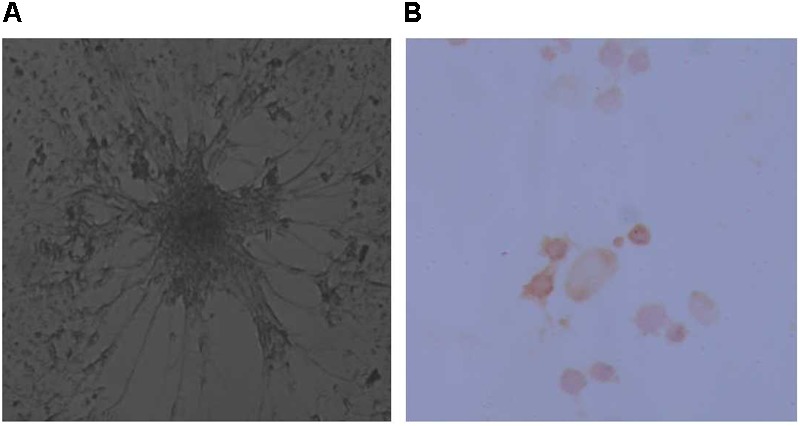
Image showing differentiation of neurospheres as evidenced morphologically **(A)** and through staining by Tyrosine Hydroxylase **(B)** to show the ability to derive dopaminergic neurons from the rat progenitor cells.

In this context, the present work revealed that neurospheres in the control group have distinct neuronal morphology with fasciculation of neurites that radiate from the central aggregation of neuronal perikarya. Paralleled to those morphological clues of differentiation, control cells demonstrate their ability to differentiate into dopaminergic neurons (15% of cells) as seen in **Figure 2**. On the other hand, TCE treated cells failed to show this differentiation capacity.

The increased size of the neurospheres in the control group is likely due to the differentiation of the cortical neural progenitor cells into mature nerve cells, which then extend axons and dendrites leading to the increased diameter of the neurospheres with the progress of time (Choi et al., 2013). This was again confirmed by differentiation of 15% of cells into dopaminergic neurons compared to the TCE treated cells which fail to differentiate.

Several mechanisms of TCE-induced neurotoxicity are suggested. For instance, Grandjean and Landrigan (2014) explained the arrest of proliferation and failure of neurogenic differentiation by decreased neurotrophic factors (Grandjean and Landrigan, 2014) which are important mediators for these processes and disturbance of these factors is claimed to be involved in neurodevelopmental disorders of the CNS (Sajdel-Sulkowska et al., 2011).

In addition, Blossom et al. (2013) observed that the abnormal behavior and neurotoxic effects in mice exposed to TCE at doses of 0.01 and 0.1 mg/ml in water could be due to oxidative stress with global DNA hypomethylation (Blossom et al., 2013).

Our second objective in the present work is to assess cytotoxicity in the neuroprogenitor cell culture exposed to TCE. It is documented that flow cytometry is the technique of choice to assess viability, apoptosis, and/or necrosis on a single cell basis (Galluzzi et al., 2015). Generally, the scatter analysis of the cell population allows a sufficient distinction between viable and non-viable cells. Additionally, staining by Annexin V and propidium iodide (PI) which is a DNA-binding dye that does not penetrate the intact cell membrane could be beneficially combined with the scatter method to recognize various types of cells. Annexin-V-positive and PI-negative cells are considered as early apoptotic while the double positive cells are classified as late apoptotic while the necrotic cells are Annexin negative and PI positive (Wlodkowic et al., 2010; Stepanek et al., 2011).

**FIGURE 3:**
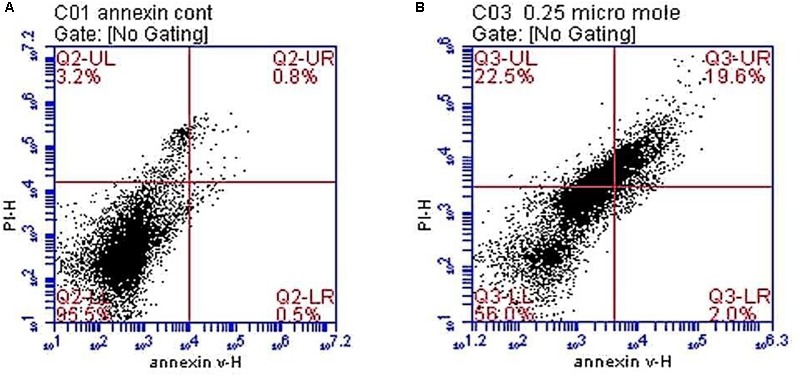
Histograms showing flowcytometric analysis of the neuronal cell cultures in control and test groups. **(A)** A histogram showing that most of the neurospheres are viable in the control group. **(B)** A histogram showing decreased cellular viability, increased necrosis, early and late apoptosis in neurospheres exposed to trichloroethylene (TCE) 0.25 μM [viable cells in the lower left quadrant (LL), early apoptotic cells in the lower right (LR) quadrant, late apoptosis is seen in the upper right (UR) while necrosis is shown in the upper left (UL) quadrants].

**Figure 3** and **Table 2** demonstrate the results of flow cytometric analysis of the cultured neurospheres. It is observed that cell viability (both Annexin V and PI negative) is significantly lower in the neurospheres exposed to TCE (0.25 μM) than that of the control cells. This finding is supported by the report of Zhu et al. (2005) who found that the normal human epidermal keratinocytes (NHEK) treated with various concentrations (0.01–31.6 mM) of TCE revealed a dose-dependent decrease in cell viability.

**Table 2:** Comparison between the studied groups regarding viability, necrosis, and apoptosis in neurospheres by flowcytometry.

Group/Parameter	Group 1 (control)	Group 2 (TCE -treated group) (TCE: 0.25 μM)	*P*-value
Viable cells	95.47 ± 0.45	57.17 ± 1.26	0.0005^∗∗^
Early apoptosis	0.36 ± 0.23	1.60 ± 1	0.2
Late apoptosis	1.5 ± 1.57	19.06 ± 1.29	0.001^∗∗^
Necrosis	2.67 ± 1.29	22.17 ± 1.04	0.00001^∗∗^

*N.B. TCE, trichloroethylene. All values were expressed as Mean ± SD.*

*Double negative cells for Annexin V and Propidium iodide (PI) = viable cells, no plasma membrane rupture, Annexin V +, but negative for PI = early apoptotic cells, Double positive cells for both Annexin V and PI = late apoptosis, Positive for PI, but negative for annexin V = necrotic cells (plasma membrane rupture).*

*P-value: ^∗∗^highly significant < 0.001.*

One of the important parameters studied in the present research is apoptosis (programmed cell death) which is a crucial process for neurodevelopment. It prevents redundant and unused neurons from disarrangement and cluttering of the developing brain (Creeley and Basavarajappa, 2016). On the other hand, necrosis had been claimed to be an unregulated mode of cell death but recently the term “necroptosis” has been used to describe a programmed, caspase-independent cell death with necrotic morphology. Its mechanism is suggested to be due to activation of the same receptors involved in apoptosis (Karch and Molkentin, 2015).

In this regard, our results reveal increased early apoptosis although it was statistically insignificant whereas late apoptosis and necrosis are significantly increased in the TCE-treated neurospheres (0.25 μM) when compared to the control cells. Accordingly, these findings observed during cell cycle analyses indicate an evident toxic effect of the examined low dose of TCE (0.25 μM) on neurospheres through induction of different types of cell death (**Figure 3** and **Table 2**).

In harmony with the present findings, an occupational study was done in lock industry workers who were exposed to TCE and its metabolites. The authors reported a significant increase of apoptosis in the collected blood samples in association with a significant up-regulation of pro-apoptotic P53 and Bax (Varshney et al., 2015). In addition, TCE exposure remarkably interferes with mitochondrial signaling through activation of caspase-dependent apoptotic cell death (McDermott and Heffron, 2013) which supports the current findings.

Furthermore, our results are in accordance with those reported by Ali et al. (2016) who studied the cytotoxic effect of low doses of TCE (0.5–32 μM) in human epidermal keratinocytes. The authors observed a significant increase in the number of cells undergoing apoptosis in the TCE-exposed groups. The findings supported the results of the present work concerning apoptosis; however, the probability of necrosis is excluded.

It is worth mentioning that apoptosis and necrosis represent two pivotal types of cell death. Apoptosis is an active process consisting of highly organized molecular events whereas necrosis is a passive uncontrolled cell rupture mediated by extremely exogenous damage. Early apoptotic cells preserve the integrity of plasma membrane to prevent the release of the potentially harmful cellular contents outside. Late apoptosis (or secondary necrosis) occurs if the early apoptotic cells are not taken up by phagocytes, which does not happen *in vitro*. Loss of the membrane integrity is a gradual process. First, the membrane of a late apoptotic cell becomes permeable for small molecules (e.g., PI) and subsequently opens also for macromolecules (Patel et al., 2006; Stepanek et al., 2011; Galluzzi et al., 2015). Accordingly, early apoptosis is insignificant at the start of the experiment but with cumulative exposure to TCE, the apoptotic process is gradually increased and the cells suffer from late apoptosis and/or necrosis due to ultimate damage of the cell membrane.

Moreover, the occurrence of necrosis in the neurospheres exposed to TCE in our work could be explained by the fact that apoptotic cells ultimately shut down metabolism after a long period of *in vitro* culture (Riss and Moravec, 2004) and exhibit some morphological forms associated with necrosis. These cells became secondary necrotic in the absence of phagocytosis and could develop features of primary necrosis (VandenBerghe et al., 2013). Additionally, numerous cell culture models and diverse study designs could also contribute to the controversial findings in various studies.

Interestingly, extrapolation of the current observations to humans exposed to TCE could be problematic. It is worth to mention that previous studies estimating serum TCE levels in human beings were highly variable due to different populations, occupational versus non-occupational exposure and various measurement methodologies. For example, in an occupational study in the United States, TCE has been estimated in the blood of 157 metal workers who had an average concentration of 2.5 μg/L (range: 0–22 μg/L) (Pfaffenberger et al., 1984). On the other hand, analysis of TCE levels in samples taken from 290 subjects revealed that the mean concentration was 0.013 μg/L besides that 88% of samples were found to be below the limit of detection (Jia et al., 2012).

As previously mentioned, pharmacokinetic modeling of TCE exhibits complicated conversion of *in vivo* to *in vitro* concentrations (U.S. Environmental Protection Agency [U.S. EPA], 2011). We used a much lower TCE concentration in the present study compared to our previously published work (Abdraboh et al., 2017), however, the used dose (0.25 μM which is equivalent to 32.85 μg/L) is still higher than the reported human serum concentrations (Pfaffenberger et al., 1984; Jia et al., 2012).

## Conclusion

The present work confirmed the potential neurotoxic effects of a very low dose of TCE (0.25 μM) in 3D neurosphere model through the affection of neural proliferation, neurite outgrowth, and differentiation in addition to disturbance of cell viability and induction of apoptosis and necrosis. The neurotoxicity of this dose of TCE which is relevant to the daily life exposure of humans to this ubiquitous pollutant is alarming and necessitates more detailed research on much lower concentrations than that investigated in the current study.

## Author Contributions

MS: concept and study design. MS and DE-N: practical work and data acquization. MS, RA-R, and SE: data analysis. MS, DE-N, RA-R, and SE: writing manuscript.

## Conflict of Interest Statement

The authors declare that the research was conducted in the absence of any commercial or financial relationships that could be construed as a potential conflict of interest.
